# Chimpanzee Adenovirus Vaccine Provides Multispecies Protection against Rift Valley Fever

**DOI:** 10.1038/srep20617

**Published:** 2016-02-05

**Authors:** George M. Warimwe, Joseph Gesharisha, B. Veronica Carr, Simeon Otieno, Kennedy Otingah, Danny Wright, Bryan Charleston, Edward Okoth, Lopez-Gil Elena, Gema Lorenzo, El-Behiry Ayman, Naif K. Alharbi, Musaad A. Al-dubaib, Alejandro Brun, Sarah C. Gilbert, Vishvanath Nene, Adrian V. S. Hill

**Affiliations:** 1 The Jenner Institute, University of Oxford, Oxford, UK; 2 Centre for Research in Therapeutic Sciences and, Institute for Healthcare Management, Strathmore University, Nairobi, Kenya; 3 International Livestock Research Institute (ILRI), Nairobi, Kenya; 4 The Pirbright Institute, Pirbright, Woking, UK; 5 Centro de Investigación en Sanidad Animal, Instituto Nacional de Investigación y Tecnología Agraria y Alimentaria (INIA-CISA), Madrid, Spain; 6 Qassim University, Qassim, Saudi Arabia; 7 King Abdullah International Research Center, Riyadh, Saudi Arabia

## Abstract

Rift Valley Fever virus (RVFV) causes recurrent outbreaks of acute life-threatening human and livestock illness in Africa and the Arabian Peninsula. No licensed vaccines are currently available for humans and those widely used in livestock have major safety concerns. A ‘One Health’ vaccine development approach, in which the same vaccine is co-developed for multiple susceptible species, is an attractive strategy for RVFV. Here, we utilized a replication-deficient chimpanzee adenovirus vaccine platform with an established human and livestock safety profile, ChAdOx1, to develop a vaccine for use against RVFV in both livestock and humans. We show that single-dose immunization with ChAdOx1-GnGc vaccine, encoding RVFV envelope glycoproteins, elicits high-titre RVFV-neutralizing antibody and provides solid protection against RVFV challenge in the most susceptible natural target species of the virus-sheep, goats and cattle. In addition we demonstrate induction of RVFV-neutralizing antibody by ChAdOx1-GnGc vaccination in dromedary camels, further illustrating the potency of replication-deficient chimpanzee adenovirus vaccine platforms. Thus, ChAdOx1-GnGc warrants evaluation in human clinical trials and could potentially address the unmet human and livestock vaccine needs.

RVFV, a negative-stranded RNA virus in the *Bunyaviridae* family, is listed as an emerging zoonotic Category A viral pathogen in the National Institute for Allergy and Infectious Diseases (NIAID) list of priority pathogens for biodefense research. The disease, Rift Valley Fever, has serious implications for livestock agriculture and trade and is also listed as a notifiable disease by the World Organization for Animal Health (OIE). Although primarily restricted to Africa, the virus can be transmitted by at least ten mosquito species that are more widely distributed than RVFV leading to concerns of disease spread^1^, as has occurred in the Arabian Peninsula and Madagascar^2^,^3^. Humans can also get infected through contact with virus-contaminated tissues and fluid^4^. Due to its epizootic nature related to heavy rainfall and flooding^5^, Rift Valley Fever is a difficult disease to study. It is thought that successive and overlapping swarms of different mosquitos infect and amplify infection rates in ruminants with subsequent transmission to humans, resulting in epidemics^6^. The high levels of human morbidity and mortality during the last major outbreak in 2006/7 in eastern Africa underscores the urgency of developing comprehensive surveillance, response and control programs, especially since there is growing evidence for inter-epidemic transmission of RVFV.

Rift Valley Fever causes high rates (>90%) of mortality in young ruminants, primarily sheep, goats and cattle. Although older animals are more resistant to disease, high rates of abortion (so-called ‘abortion storms’) are observed following RVFV infection in pregnant animals and this is often used as a warning sign of imminent human disease epidemics^7^. Unlike other domestic ruminants, RVFV infection in dromedary camels tends to be mild or inapparent, with abortion among pregnant animals being the only clinical sign^8^. However, severe clinical signs, including haemorrhagic septicaemia and sudden death, have been observed among infected dromedary camels in Mauritania^9^. In humans RVFV infection presents as an acute self-limiting febrile illness, but severe manifestations, including haemorrhagic fever and encephalitis, also occur, with case fatality rates >30% reported in some outbreaks, and long-term sequelae (e.g. impaired vision) in some survivors^10^,^11^. Live and inactivated RVFV vaccines are available for livestock, but no licensed vaccines or anti-viral therapies are currently available for humans.

Recovery from natural RVFV infection results in long-lived cross-strain immunity conferred by neutralizing antibodies against the viral envelope glycoproteins, Gn and Gc, which are conserved across different viral isolates^12^,^13^. Thus, whilst the neutralizing antibody titre threshold required for protection against RVFV infection is currently unknown, development of vaccines that elicit antibody titres within the range induced by natural infections is a very attractive way forward. The widely used live attenuated RVFV vaccines (e.g. Smithburn vaccine) in livestock in Africa^14^ elicit high titre neutralizing antibody and provide durable cross-strain protection. However, these livestock vaccines carry the risk of reversion to virulence and risks of teratogenicity and abortion^14^, making their general use in humans very unlikely. A formalin-inactivated investigational RVFV vaccine, TSI-GSD-200, has previously been evaluated in humans and found to be safe but poorly immunogenic, requiring three primary immunizations and a booster dose to generate and maintain neutralizing antibody responses^15^. A live-attenuated RVFV vaccine has undergone clinical testing and the study registered as completed in 2012 but results are as yet unpublished (ClinicalTrials.gov No. NCT00415051). Development and licensure of safe, highly immunogenic and efficacious RVFV vaccines for humans is clearly an unmet need for this neglected public health threat. The use of vaccine platforms with a well-established human safety profile is an attractive strategy for this purpose.

Replication-defective chimpanzee adenoviruses (ChAd) are among the most promising human vaccine platforms available. Unlike platforms that utilize adenoviruses to which humans are naturally exposed to (e.g. HAdV5) ChAd vectors are not prone to significant anti-vector immunity that could limit vaccine performance in humans^16^,^17^. In addition, ChAd vectors have a large insert capacity (~8 kb), achieve high level, persistent transgene expression and are adaptable for use with diverse immunogens. Of relevance to their potential use in a human RVFV vaccine, ChAd vectors, including ChAdOx1^17^, have passed safety evaluations in humans for a wide range of infectious disease targets including malaria^18^, HIV^19^, tuberculosis, influenza^20^, hepatitis C^21^, RSV^22^ and, most recently, Ebola^23^. Their use as a common vaccine development platform has the advantage of allowing multiple vaccines to be biomanufactured rapidly with standardized processes and low cost of goods.

We previously showed that a single-dose immunization with ChAdOx1-GnGc, composed of ChAdOx1^17^,^20^, a species E adenovirus, encoding RVFV Gn and Gc, elicits high-titre neutralizing antibody and confers protection against lethal viral challenge in mice^24^. Here, to further evaluate ChAdOx1-GnGc as a potential candidate vaccine for humans and livestock we determined its immunogenicity and protective efficacy against virulent RVFV challenge in sheep, goats and cattle in a disease-endemic setting in Kenya. In addition, we evaluated the immunogenicity of ChAdOx1-GnGc in dromedary camels in Saudi Arabia and compared vaccine-elicited responses to those measured in the sheep, goat and cattle efficacy studies in Kenya.

## Results and Discussion

Sheep (Dorper breed), goats (Galla breed) and cattle (Holstein-Friesian breed) aged 4–6 months were sourced from local commercial farms and pre-screened for previous exposure to RVFV using the serology-based ID Screen® RVF kit (IDvet, France). Groups of seronegative animals were then immunized intramuscularly on the right base of the neck with 10^9^ infectious units (iu) ChAdOx1-GnGc without adjuvant (group 1), 10^9^ iu ChAdOx1-GnGc mixed with the saponin-based Matrix-Q^TM^ adjuvant (group 2) or placebo immunized with phosphate buffered saline (PBS; group 3). A fourth comparator group received the licensed live Smithburn RVFV livestock vaccine widely used in Africa (group 4). Four weeks later all animals were challenged by subcutaneous inoculation of 10^7^ plaque-forming units of the same batch of a heterologous virulent RVFV strain 56/74IN^25^ and monitored for 14 days after which they were culled. The primary endpoint for efficacy was development of viraemia as measured by qRT-PCR^26^ in whole blood sampled daily over the monitoring period.

**Figure 1:**
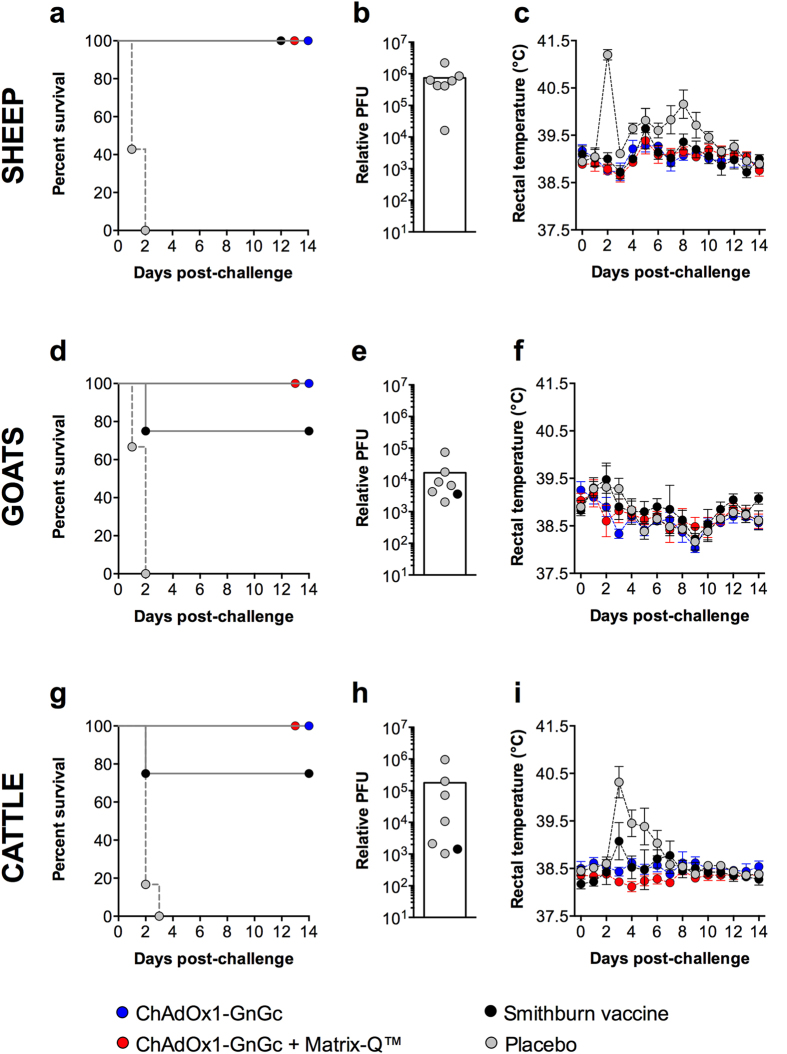
ChAdOx1-GnGc vaccination protects sheep, goats and cattle against RVFV challenge. Kaplan-Meier plots are used to infer vaccine-mediated protection using the primary endpoint of qRT-PCR detection of viraemia over a 14-day period following challenge (**a**, sheep; **d**, goats; **g**, cattle). Peak viraemia levels for each species are shown as relative plaque-forming units (pfu; bars represent means), estimated by extrapolation from a standard curve generated using serial dilutions of RNA isolated from the challenge virus and assayed using the same method as the test samples^26^ (**b**, sheep; **e**, goats; **h**, cattle). Rectal temperature data measured at the same time of day during post-challenge monitoring are shown by vaccine allocation (**c**, sheep; **f**, goats; **i**, cattle), presented as means and standard errors. The respective group sizes for each species are detailed in the Methods section.

Whilst all sheep, goats and cattle in the placebo group developed viraemia, no viraemia could be detected among any of the ChAdOx1-GnGc vaccinees in all three species (Fig. 1). Marked increase in rectal temperature accompanied viraemia in sheep and cattle (Fig. 1c,i), but not in goats (Fig. 1f) confirming the unreliability of rectal temperature as a read-out for vaccine efficacy in this species^7^,^27^. The licensed Smithburn vaccine was also protective but, consistent with previous reports of its variable immunogenicity in some livestock^14^,^28^, one goat and one calf receiving this vaccine developed viraemia post-challenge (Fig. 1e,h).

**Figure 2:**
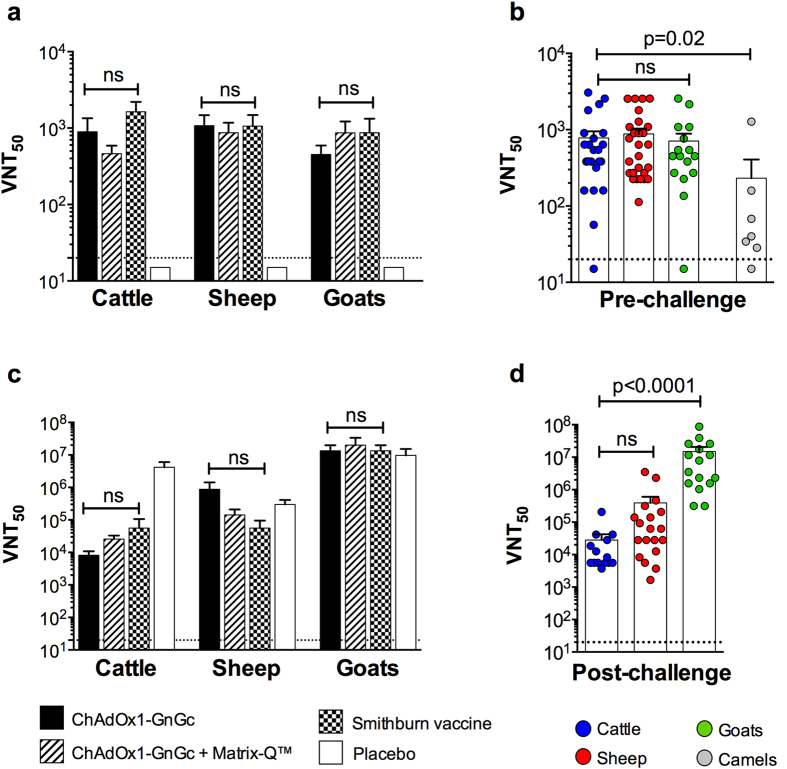
ChAdOx1-GnGc elicits high-titre RVFV neutralizing antibody. For each species means and standard errors of RVFV neutralizing antibody titres measured 28 days post-vaccination are shown in (**a**) and titres measured 14 days post-challenge are shown in (**c**). Pooled pre-challenge (**b**) and post-challenge (**d**) neutralizing antibody data from vaccinees (ChAdOx1-GnGc groups and Smithburn group) are shown, with each point representing an animal and bars representing the means and standard errors. Neutralizing antibody titres measured 28 days post-ChAdOx1-GnGc immunization in dromedary camels are shown in (**b**). All analyses are by the Kruskal-Wallis test, with Dunn’s correction for multiple comparisons between groups. The respective group sizes for each species are detailed in the Methods section. ns –not statistically significant (p > 0.05). The dashed line represents the detection limit of the assay.

**Figure 3:**
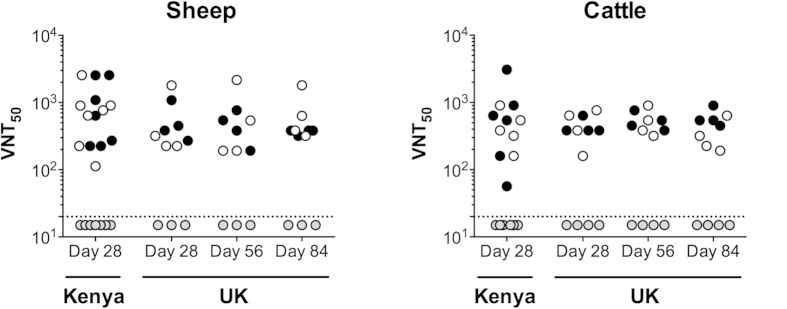
ChAdOx1-GnGc elicits comparable RVFV neutralizing antibody titres in Kenyan and UK livestock. Sheep and cattle in the UK were immunized with either ChAdOx1-GnGc (n = 4/species), ChAdOx1-GnGc with Matrix-Q™ (n = 4/species) or placebo (n = 3 sheep, n = 4 cattle) using the same vaccine dose and volumes as in studies in Kenya (see Methods). RVFV neutralizing antibody titres were then measured over a 3-month period before culling. As in the Kenyan studies, Matrix-Q™ did not enhance the magnitude of the response in either species across the study period and the range of titres across time points were comparable to those in sheep (Kruskal-Wallis test p = 0.6) and cattle (Kruskal-Wallis test p = 0.9) in Kenya. Black circles–ChAdOx1-GnGc, clear circles–ChAdOx1-GnGc with Matrix-Q™, grey circles–placebo. The dashed line represents the detection limit of the assay.

Next we measured RVFV neutralizing antibody titres in sera sampled four weeks post-immunization. In all three species ChAdOx1-GnGc vaccination elicited high-titre neutralizing antibody, comparable to the licensed Smithburn vaccine (Fig. 2a). Similar neutralizing antibody titres were observed in parallel ChAdOx1-GnGc immunogenicity studies in sheep and cattle in the UK (Fig. 3), and remained high at 3 months post-vaccination. We could not detect neutralizing antibody in sera from the viraemic Smithburn vaccinees (Fig. 1e,h). Further, unlike studies utilizing a similar adjuvant in mice^24^, Matrix-Q™ adjuvant had no effect on the antibody response elicited by ChAdOx1-GnGc in any of the livestock species (Figs 2a and 3), highlighting the importance of evaluating candidate vaccine regimens in target species.

Significant post-challenge boosting of neutralizing antibody titres was observed in all three species independent of vaccine allocation (Fig. 2c), though overall responses were notably higher among goats. To perform inter-species comparisons of the magnitude of boosting, data from vaccinees were pooled, maximizing statistical power whilst minimizing the number of comparisons. As expected, pooled pre-challenge neutralizing antibody titres were comparable between species (Fig. 2b). However, post-challenge antibody titres were significantly higher in goats compared to sheep and cattle (Fig. 2d) suggesting potential differences in the kinetics and/or nature of secondary adaptive immune responses between species.

Next, given their susceptibility to RVFV infection and role in virus transmission^8^,^9^ we sought to evaluate ChAdOx1-GnGc immunogenicity in dromedary camels. We sourced dromedary camels aged 1 to 2 years from commercial farms in Qassim, Saudi Arabia and screened for previous exposure to RVFV by virus neutralizing antibody assay. Eleven unexposed camels were then randomly allocated into two groups for intramuscular immunization with 10^9^ iu ChAdOx1-GnGc (n = 7), as in the efficacy studies in Kenya, or placebo (PBS, n = 4). Six of the seven ChAdOx1-GnGc vaccinees mounted a neutralizing antibody response detectable at day 28 post-vaccination (Fig. 2b) and the remaining ChAdOx1-GnGc vaccinee had seroconverted by the next sampling time point (day 56 post-vaccination). Neutralizing antibody titres between the two time points were comparable (mean at day 28 = 70, range 0–1277 vs. mean at day 56 = 108, range 40–384; Wilcoxon matched pairs signed rank test p = 0.7). However, the titres elicited in camels were lower in comparison to those observed in sheep, goats and cattle at the same ChAdOx1-GnGc dose and time post-vaccination (day 28; Kruskal-Wallis test p = 0.02; Fig. 2b), but still within the range associated with protection in other livestock studies^29^,^30^. However, lack of appropriate biocontainment facilities for camel RVFV challenge studies limited our ability to directly determine the protective efficacy of this response, though this is clearly a priority for future studies.

In summary we have demonstrated the utility of the replication-deficient chimpanzee adenovirus platform in induction of functional antibody and protective immunity against RVFV in multiple target livestock species in a disease-endemic setting. Larger field efficacy and dose optimization studies of ChAdOx1-GnGc in animals of different age groups and physiological status will be required to underpin its future licensure and general use in livestock, and to explore potential inter-species differences in vaccine-elicited immune responses. The durability of the immune response in vaccinees, as well as safety and efficacy in pregnant animals and prevention of mortality in neonatal animals will be key endpoints in such studies. The current widely used livestock RVFV vaccines have major safety concerns^14^ and their use makes it impossible to distinguish infected from vaccinated animals (DIVA) since the anti-RVFV antibody profile in both groups of animals is similar. DIVA compatibility is particularly important for livestock disease surveillance and rapid effective control during epizootics. ChAdOx1-GnGc, like other candidate vaccines in development^31^,^32^, only encodes RVFV envelope glycoproteins-the major target of protective immunity - making it compatible with commercially available serological DIVA kits that assign ‘vaccinated’ (only seropositive for RVFV envelope glycoproteins) or ‘infected’ (seropositive for all RVFV antigens) status with high sensitivity and specificity.

Evaluation of ChAdOx1-GnGc in human clinical trials is an obvious next step. However, because deliberate human infection with RVFV to test vaccine efficacy is not possible, the pathway to licensure for human use will likely rely on special approval processes such as the US Food and Drug Administration Animal Efficacy Rule in which licensure for an intervention with an acceptable safety profile can be obtained based on its protective efficacy in suitable animal species. Evaluation of the most promising human RVFV vaccine candidates in clinical trials to support licensure will ensure better preparedness for future outbreaks through vaccine stockpiling, allowing rapid deployment in first response teams of health workers and others at most risk of RVFV infection. This is now a favoured approach for high impact outbreak pathogens in general, and is among key lessons emerging from the devastating Ebola virus disease outbreak in West Africa^33^.

Finally, the One Health vaccine development approach used here, by which the same vaccine is co-developed for humans and susceptible animal species, is well suited to many emerging outbreak pathogens, most of which involve zoonotic transmission^34^. Further to providing stringent evaluation of a candidate human vaccine in pre-clinical efficacy studies utilizing actual target animal species, the approach allows the possibility of cost reductions for the final product by increasing the scale of manufacture. GlaxoSmithKline, one of the largest pharmaceutical companies, is already developing large-scale biomanufacture capacity for replication-deficient chimpanzee adenovirus vaccines indicating strong industrial interest for the platform^23^.

## Methods

### Vaccines, adjuvant and RVFV

The ChAdOx1-GnGc vaccine was prepared by Gateway® recombination between the ChAdOx1 destination vector^17^ and an entry plasmid containing the coding sequence for RVFV Gn and Gc (Genbank accession number DQ380208, bases 411–3614) as described (22). The live attenuated Smithburn RVFV vaccine (Riftvax™) was obtained from the Kenya Veterinary Vaccines Production Institute, Kenya, for use as a comparator in the efficacy experiments. The saponin-based Matrix-Q™ adjuvant, developed and licensed for use in ruminants, was obtained from Novavax AB, Sweden. The virulent RVFV strain 56/74IN was propagated in C6/36 insect cells (23), purified by ultracentrifugation and resuspended in PBS for challenge of sheep, goats and cattle as outlined below.

### RVFV challenge experiments in Kenya

All animal challenge experiments were reviewed and approved by the International Livestock Research Institute’s Institutional Animal Care and Use Committee (ILRI-IACUC) and conducted in accordance with the ILRI-IACUC and the Kenya National Biosafety Authority guidelines. Four to six-month old sheep, goats and cattle were sourced from local commercial farms and pre-screened for previous exposure to RVFV by the ID Screen® RVF kit (IDvet, France). Sheep were of the Dorper breed, cattle of the Holstein-Friesian breed and goats of the Galla breed. Following an acclimatization period in the animal facility, animals were randomly allocated to four experimental groups and vaccinated intramuscularly at the right base of the neck with 10^9^ infectious units (iu) ChAdOx1-GnGc without adjuvant (group 1; n = 7 sheep, 6 goats, 6 cattle) or 10^9^ iu ChAdOx1-GnGc mixed with 100 μg (sheep and goats) or 400 μg (cattle) Matrix-Q^TM^ adjuvant (group 2; n = 7 sheep, 6 goats, 5 cattle) or placebo-vaccinated with phosphate buffered saline (PBS, group 3; n = 7 sheep, 6 goats, 6 cattle). All immunizations were done in a total volume of 1 ml. A fourth group of 4–5 animals was vaccinated with the Smithburn vaccine as per manufacturer’s instructions, and used as positive controls (group 4; n = 5 sheep, 4 goats, 4 cattle).

Serum was sampled from all animals on the day of vaccination (day 0) and on day 28 post-vaccination. The immunogenicity endpoint for this study was the induction of RVFV neutralizing antibody, measured pre-challenge (day 28 post-vaccination). All animals were challenged by subcutaneous inoculation of 10^7^ plaque-forming units of RVFV 56/74IN on the left base of the neck on day 28 post-vaccination and monitored for the next 14 days. The subcutaneous route was chosen as one that consistently induces viraemia in ruminants^7^,^27^. The primary efficacy endpoint was viraemia as measured by qRT-PCR in whole blood sampled daily over the entire two-week monitoring period. The qRT-PCR assays were done on a StepOnePlus™ Real-Time PCR system using TaqMan® Fast Virus 1-Step Master Mix with published primers, probe and cycling conditions^26^. The group sizes of ≥4 provided 90% power for a two-sample comparison of proportions at an alpha of 5%, allowing detection of 100% efficacy in the vaccine groups (i.e. no viraemia post-challenge) versus no protection in the placebo group (i.e. presence of viraemia post-challenge in all animals). All animal staff and investigators were blinded to group allocation during the experiment and measurement of efficacy and immunogenicity endpoints.

### ChAdOx1-GnGc immunogenicity studies in the UK and Saudi Arabia

In parallel with studies in Kenya ChAdOx1-GnGc immunogenicity was evaluated in Merino sheep and Holstein-Friesian cattle at the Pirbright Institute, UK, having been reviewed and approved by the institute animal ethics committee. The study was conducted in accordance with the UK Home Office guidelines. Two to four-month old lambs and calves were sourced from commercial farms. For each species, groups of animals each were immunized with 10^9^ iu ChAdOx1-GnGc without adjuvant (n = 4 sheep, 4 cattle) or 10^9^ iu ChAdOx1-GnGc co-administered with Matrix-Q™ (n = 4 sheep, 4 cattle) as in the studies in Kenya. Placebo controls (n = 3 sheep, 4 cattle) received PBS. Serum was sampled from all animals on the day of vaccination (day 0) and on days 28, 56 and 84 post-vaccination after which animals were culled.

Following on from the Kenya and UK studies we evaluated the immunogenicity of ChAdOx1-GnGc in dromedary camels, sourced locally in Qassim, Saudi Arabia and pre-screened for previous exposure to RVFV by neutralizing antibody assay. Eleven unexposed camels were acclimated to the animal facility at Qassim University college of Agriculture and Veterinary Medicine and randomly allocated to intramuscular 10^9^ iu ChAdOx1-GnGc immunization (n = 7) or placebo (PBS, n = 4). Serum was sampled from all animals on the day of vaccination (day 0) and on days 28 and 56 before culling. All experimental procedures were conducted in accordance with the institutional animal use guidelines at Qassim University.

### Assessment of RVFV neutralizing antibody responses

Sera were heat-inactivated for 30 minutes at 56 °C before use. Then, serial two-fold dilutions of the sera were made in 100 μl Dulbecco’s Modified Eagle Medium containing 10% fetal bovine serum (DMEM-10) to which a further 100 μl of DMEM-10 containing 100 TCID_50_ of the MP-12 RVFV strain was added. After a one-hour incubation at 37 °C the virus-serum mixture was transferred onto confluent Vero cell monolayers in 96-well plates in quadruplicate and incubated for 72 hours at 37 °C in a 5% CO_2_ incubator. The cells were then fixed in PBS containing 10% formaldehyde, stained in 1% crystal violet, plaque formation scored and the Spearman-Karber algorithm used to determine the 50% endpoint titres (hereafter termed VNT_50_).

### Statistical analyses

All analyses were performed using non-parametric tests in GraphPad Prism® v6 using a two-sided p value <0.05 as the cut-off for statistical significance.

## Additional Information

**How to cite this article**: Warimwe, G. M. *et al.* Chimpanzee Adenovirus Vaccine Provides Multispecies Protection against Rift Valley Fever. *Sci. Rep.* **6**, 20617; doi: 10.1038/srep20617 (2016).
